# Differential Haemoparasite Intensity between Black Sparrowhawk (*Accipiter melanoleucus*) Morphs Suggests an Adaptive Function for Polymorphism

**DOI:** 10.1371/journal.pone.0081607

**Published:** 2013-12-31

**Authors:** Bonnie Lei, Arjun Amar, Ann Koeslag, Tertius A. Gous, Gareth J. Tate

**Affiliations:** 1 Percy FitzPatrick Institute of African Ornithology- DST/NRF Centre of Excellence, University of Cape Town, Cape Town, South Africa; 2 Department of Organismic & Evolutionary Biology, Harvard University, Cambridge, Massachusetts, United States of America; 3 Specialist Veterinary Pathology, Helderberg, South Africa; University of Lausanne, Switzerland

## Abstract

Recent research suggests that genes coding for melanin based colouration may have pleiotropic properties, in particular conveying raised immune function. Thus adaptive function of polymorphism may be associated with parasite resistance. The black sparrowhawk *Accipiter melanoleucus* is a polymorphic raptor with two morphs. Over most of its range the light morph is commonest, however within the recently colonised Western Cape of South Africa the dark morph predominates. The species breeds in winter throughout South Africa, however unlike in the rest of the species' South African range, the Western Cape experiences a winter rainfall regime, where arthropod vectors which transmit haematozoan parasites may be more abundant. We hypothesise that the higher frequency of dark morph birds in this region may be due to their improved parasite resistance, which enables them to cope with higher parasite pressure. If so, we predict that dark morph black sparrowhawks would have lower parasite burdens than light morph birds. Within our population the prevalence of the two most common haematozoan parasites was high, with 72% of adults infected with *Haemoproteus nisi* and 59% of adults infected with *Leucocytozoon toddi*. We found no difference in prevalence for either parasite between adult morphs, or between chicks of different parental morphs. However, within adults infected with *H. nisi*, infection intensity was significantly higher in light morphs than dark morphs. This suggests that dark morphs have lower parasite loads than light morphs due to resistance rather than morph-specific habitat exploitation. Greater resistance to *Haemoproteus* parasites may therefore be one of the mechanisms through which dark morph black sparrowhawks have a selective advantage in this region and may explain why they are most common in our study area. In other regions, the cost to benefit ratio may be in favour of the light morph, where parasites are less abundant or virulent.

## Introduction

Persistent colour polymorphism occurs when two or more distinct and genetically determined colour morphs are present within a single population at frequencies too great to be maintained purely by recurrent mutation [1]. For many polymorphic species, the pigment melanin is responsible for the colour variations in morphs [1]–[3]. Numerous studies have found that melanin-based colour morphs frequently show different life-history traits, and this finding has led to the hypothesis that different morphs are adapted to different environments, which may vary in their levels of food abundance, social interactions or parasite exposure [1]. Although many studies have suggested an adaptive function for polymorphisms, very few have identified the selective agent or mechanisms involved [4].

It has been suggested that the gene, melanocortin-1 receptor (MC1R), which commonly codes for melanin production in many animals [5]–[7] also controls expression of aspects of immune function that might be important for parasite resistance [8]–[13]. Thus, darker individuals gain a selective advantage in areas or conditions where parasites entail larger fitness costs. If so, variations in MC1R expression might correlate not only with plumage or pelage colour polymorphisms, but also with immune function.

In support of such pleiotropic properties, recent research has suggested that parasite loads may differ between colour morphs in a number of taxa. For example, Atlantic salmon *Salmo salar* with more black, melanin-based, skin spots were found to have fewer female sea lice with egg sacs, compared to less pigmented individuals [14]. For birds, [15] found that more heavily spotted female barn owls *Tyto alba* had nestlings with lower ectoparasitic fly burdens and studies on tawny owls *Strix aluco* have demonstrated that rufous birds have more blood parasites than grey birds [16]. More recently, [17] demonstrated that darker pigeons *Columba livia* had lower Haemosporidian parasite infection intensity than paler birds. In the only study to explore this issue in a raptor species, [18] similarly found that infection intensity of the blood parasite *Leucocytozoon toddi* was lower in dark morph common buzzard *Buteo buteo* nestlings as compared with intermediate and pale nestlings, but that prevalence was lower in offspring of intermediately coloured males. In contrast, [19] failed to find strong differences in a range of parasites between the banaquit morphs on the island of Grenada, including the prevalence of haematazoan blood parasites. Although unlike the other studies, [19] only examined prevalence and not infection intensity. Furthermore, several studies have shown relationships between an individual's immune response and their colour morph or parental morph [6], [18], [20]. Thus from the limited studies so far carried out, there is a strong suggestion that immune capacity and parasite infections may differ between morphotypes in polymorphic species.

In theory, these studies suggest that differential resistance to parasites could be an important mechanism in the maintenance of colour polymorphisms [17], [18]. For example, dark morphs with higher resistance to a certain damaging parasite may have a selective advantage in areas or habitats where that parasite is more abundant, whereas in other locations with lower parasite pressure, the lighter morphs may be favoured, perhaps due to the increased costs of being dark or mounting an unnecessarily high immune response [20], or due to contrasting selection pressures exerted by other parasites [18]. This concept of local adaptation, or the maintenance of phenotypic variation in species by differential selection in different environmental conditions, has been suggested as the mechanism for the maintenance of colour polymorphism or clines in several bird species. Comparative analysis suggests that in owls, nightjars and raptors, polymorphism was most common in species with the widest niche breadth [21]. In barn owls (*Tyto alba*) local adaptation through habitat selection and improved reproductive output, appears important in maintaining the cline in phenomelanin colouration [22].

Among raptors, polymorphism occurs frequently (24%) in the *Accipiter* genus [23]. The black sparrowhawk *Accipiter melanoleucus* is a widely distributed *Accipiter* species inhabiting forested areas throughout much of sub-Saharan Africa [23]. Adults display discrete plumage polymorphism, occurring as either dark or light morphs. Morph type is apparently inherited in a typical Mendelian manner, that suggests a one-locus, two-allele system in which the allele coding for the light morph is dominant [24]. Within South Africa, the species has recently expanded its range and colonised the far southwest, with the first nest in the Cape Peninsula recorded in 1993 [25], [26]. Morph frequency varies clinally throughout their South African range, with the frequency of dark morphs declining from over 80% in the Cape Peninsula to under 20% in the north east [24]. As a winter breeder, the species therefore breeds in the wet season in the south west but during the dry season in the rest of its South African range [27]. Haemosporidian parasites loads tend to be higher among birds in wetter areas or at wetter times of year [28], [29] and haemoparasites are known to have a detrimental effect on reproductive success and survival in birds [30], [31]. Thus, it is possible that a selective advantage for increased defence against haemoparasites in wetter conditions may drive the clinal variation and the high frequency of dark morph birds that are present in the Cape Peninsula.

Given the known association between melanin-based polymorphism and immune function, we investigate the prevalence and infection intensity of haemosporidian parasites (*Haemoproteus* nisi and *Leucocytozoon* toddi) in relation to colour morph of black sparrowhawks on the Cape Peninsula. We seek to test the hypothesis that dark morphs have a selective advantage in our study area through an increased immune response to parasites. If true, we predict that haemosporidian parasites prevalence and/or infection intensity will be lower in dark morphs than light morphs birds. Additionally we investigate whether parasite infections differ across ages and sexes in this population and whether the parental morph influence parasite infections of their offspring.

## Materials and Methods

We monitored the black sparrowhawk population on the Cape Peninsula between 2001 and 2012, although data used in this study comes exclusively from 2009 to 2011. The study area features a matrix of habitats including urban gardens, alien pine (*Pinus* spp.) and eucalyptus (*Eucalyptus* spp.) plantations, small pockets of indigenous Afromontane forest, and Fynbos. Altitude ranges from sea level to about 300 m, and the climate is temperate, with locally variable winter rainfall [32]. Mean annual rainfall is c.1250 mm, with average minimum and maximum monthly temperatures of 12° and 21°C, respectively (South African Weather Service).

Monitoring was conducted during the breeding season (March to November; [27]) each year. Nests were located by surveying suitable stands of trees during the breeding season, searching for calling sparrowhawks, prey remains, whitewash and nest structures. Territories were visited regularly (approximately monthly) throughout the season until breeding was detected and then breeding attempts were monitored until conclusion. We identified the morphs (dark or light) and sex of both parents attending each nest, whereas nestlings do not yet express colour polymorphism, which only becomes apparent when they moult into adult plumage in their second year. The species is easy to sex, with males being around 40% lighter in mass than the females [23].

Blood samples were collected from chicks and adults in various locations throughout the Cape Peninsula between 2009 and 2011. Adults were trapped and sampled on territories using a bal-chaltri trap baited with live white pigeons (*Columba livia*) [33]. Chicks were sampled when they were 2–4½ week old nestlings. We obtained blood samples from 44 adults (25 males (17 dark : 8 light) and 19 females (15 dark : 4 light), 1 juvenile (aged 10 weeks and classed as a chick for the purpose of these analyses), and 106 chicks (67 males and 39 females). Adults were trapped on 28 territories, with multiple birds being captured on 10 territories. Chicks came from 55 broods from 36 different territories. The protocol for fieldwork was approved by the University of Cape Town's Science Faculty Animal Ethics Committee (Permit number: 2012/V37/AA). Much of the field work took place on Table Mountain National Park and approval to work in the park was provided by South African National Parks, other work took place on private property and in all cases approval to work in these areas was provided by the owners of the land.

Haemosporidians (Sporozoa: Haemosporida) are protists, which include the malarial parasites, all of which employ blood-sucking dipteran vectors to infect their amphibian, reptile, bird, or mammal hosts [34]. Within Accipiters, *Leucocytozoon spp* and *Haemoproteus spp* are the most frequently encountered haemosporidian parasites [29]. *Leucocytozoon* spp are malarial parasites exclusive to birds, using blackflies (*Simulium* species) as their definitive host and birds as their intermediate host. *Haemoproteus* spp are transmitted by blood sucking insects, and in birds this is usually the louse flies (*Hippoboscidae*) which are commonly found in bird's nests.

### Blood Smear Preparation

To collect blood samples, the skin over the cubital vein was first sterilized with an alcohol swab and the vein was then dilated with mild pressure applied proximal to the sampling point. A small puncture hole was made at the vein by means of a sterilized 21 gauge needle (BD Micro-Fine Plus, Alpha Pharm East Cape (Pty), Port Elizabeth); blood was then extracted through capillary action into 80 µL×75 mm heparinised capillary tubes (Lasec SA (Pty), Cape Town). Thin blood smears were prepared by placing a small drop of the heparinized blood at the end of a dry microscope slide, which was spread out along the slide's length. The blood smear was air dried and transferred in a slotted carrier box ensuring contact with only air during transportation. These slides were then fixed by immersion in methanol for 2 minutes prior to staining using Giemsa stain (10×, diluted with Giemsa buffer) for 2 minutes to visualize blood cells and parasites.

### Blood Smear Analysis

Slides were examined using a binocular compound microscope with oil immersion lenses. We first scanned for parasites at 500× magnification for 15–20 minutes per slide covering most of the slide during this time. All slides received the same overall search effort by the same individual (BL), and a good representation including both peripheries of the smear as well as the central areas with higher densities of erythrocytes. On average 461.9±33.0 erythrocytes were observed per field of view.

A focus was placed specifically on parasites known to afflict *Accipiters*, *Leucocytozoon toddi* and *Haemoproteus nisi* [29], [34], [35]. Once a suspected haematozoan was detected from the 500× scan, the magnification was increased to 1000× to examine the suspected cells, to confirm the infection, and to identify the haematozoa to species level, based on [36]. For each of our two haematozoa parasites we obtained two measures; 1) Prevalence - defined as the presence of any infected cell that were found on the slide, which was a binary measure (0 = uninfected; 1 = infected) for each sample and therefore for each bird. 2) Infection intensity – defined as the number of fields of view at 500× magnification where at least one infected cell was seen. Following [37], infection intensity was only analysed in infected individuals (i.e. those with a prevalence of 1).

The same person who originally examined the slides then re-sampled a random subset of 30 of the 148 slides (20%) without knowledge of their prevalence status or infection intensity. Both measures (prevalence and infection intensity) were found to be highly repeatable. In all cases, the same prevalence status was recorded in both examinations, and for infection intensity for both parasites there was a high degree of repeatability between the original and repeat examination (*H. nisi*: r = 0.57; *L. toddi*: r = 0.51) [38].

### Statistical analysis

We used Generalised Linear Models (GLiM) to explore differences in *L. toddi* and *H. nisi* prevalence and infection intensity. We first explored for differences in infection between different ages (adults and chicks). For adults we also used these same models to examine differences in parasite infection between the sexes and between the two morphotypes (dark or light) testing for each term in a univariate manner, unless otherwise stated. However, for chicks the blood samples belonging to chicks from the same brood were not independent, and therefore, to account for this lack of independence we used Generalised Linear Mixed Model (GLMM) with brood specified as a random term. Within the GLMM, denominator degrees of freedom were estimated using the Kenward-Rogers method. We used these GLMM's to test for differences in prevalence and intensity of each parasite between the sexes of chicks and also between chicks from different parental morphs, again testing for each term in a univariate manner.

In all models, we examined the difference in parasite prevalence specifying a binomial error and logit link function. For birds infected by either parasite, infection intensity (number of fields of view with an infected cell) was modelled using a Poisson error structure (corrected for extra dispersion) and a log link function. For chicks, only 12 (from 104) individuals were infected with *H. nisi*, therefore, when examining difference between different groups of chicks (eg. by sex or by parental morph) we only examined variation in infection intensity for *L. toddi*. All means are presented ±1 S.E, unless otherwise stated. Models were implemented in R version 2.15.1 (R Development Core Team 2009) or for the GLMMs using SAS version 9.1 [39].

## Results

### Age differences in parasite infections

**Figure 1:**
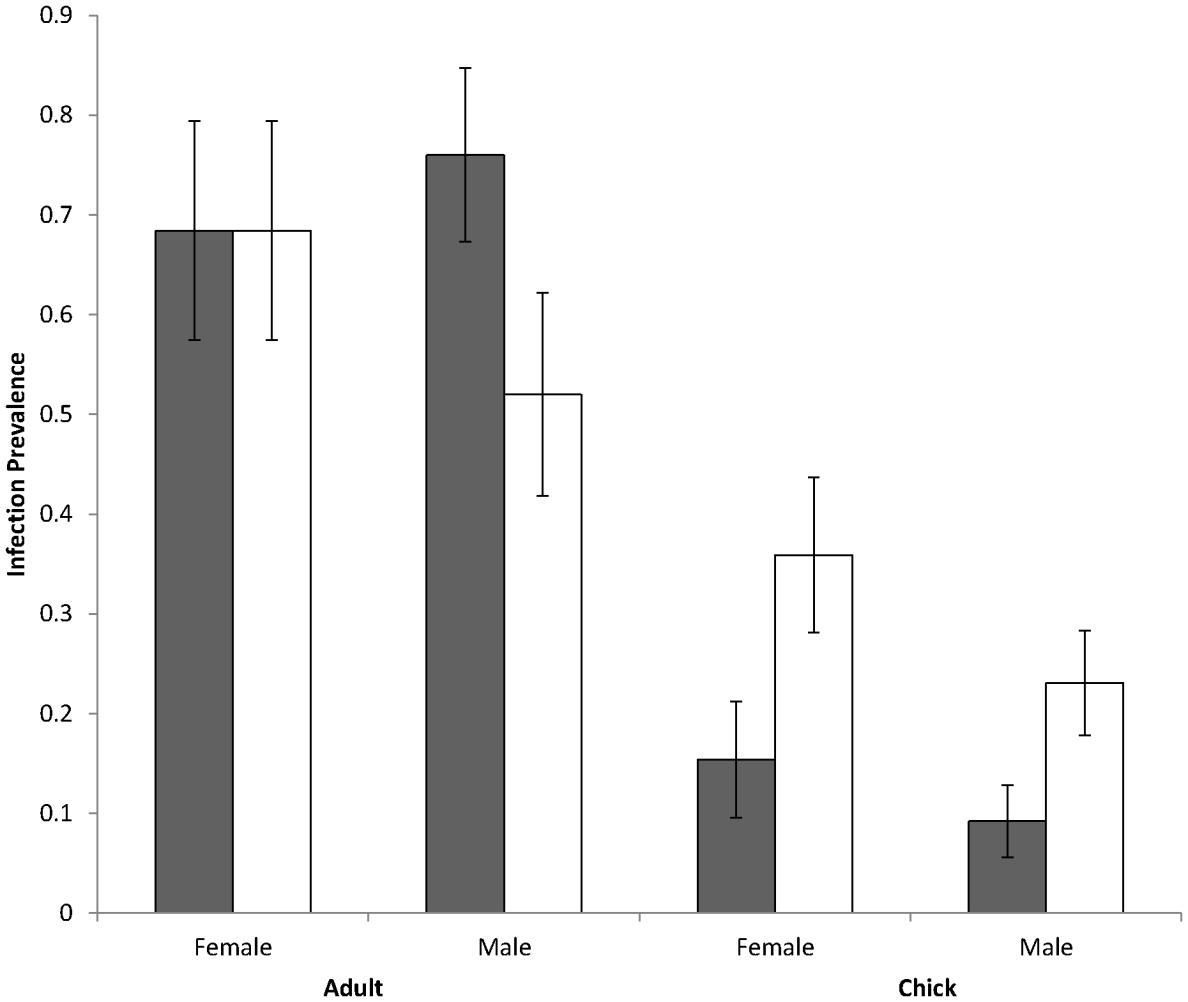
Comparisons of blood parasite *Haemoproteus* (dark bars) and *Leucocytozoon* (light bars) infection prevalence in black sparrowhawks of different age and gender.

**Figure 2:**
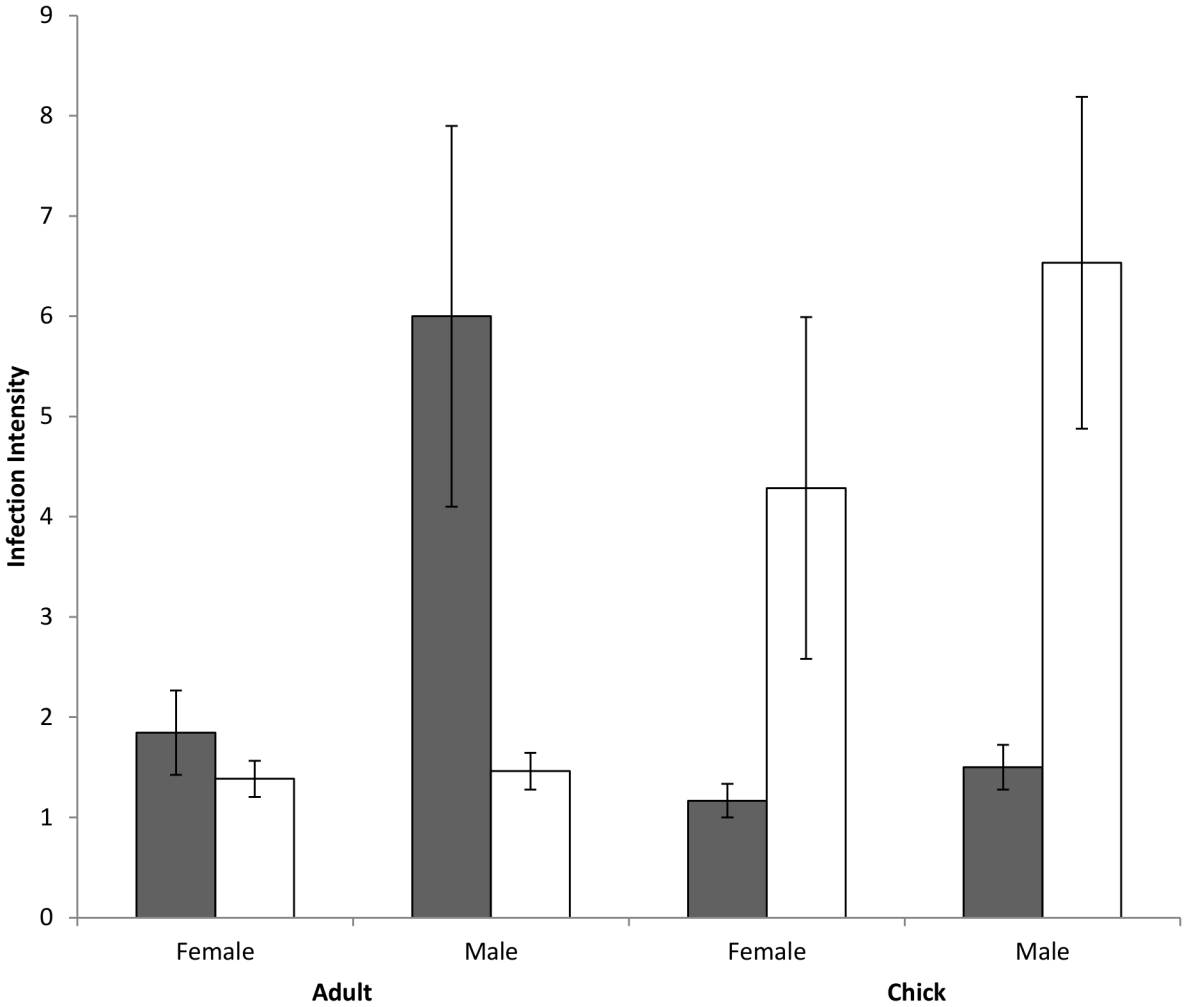
Comparisons of blood parasite *Haemoproteus* (dark bars) and *Leucocytozoon* (light bars) infection intensities in black sparrowhawks of different age and gender.

The prevalence of *H. nisi*, in adults was over six times higher than in chicks (χ^2^_1, 146_ = 54.183, *P*<0.0001; Table 1; Fig. 1). Amongst infected individuals, adults also had a higher infection intensity than chicks (*F*_1, 42_ = 5.78, *P* = 0.01; Table 1; Fig. 2). Likewise infection prevalence of *L. toddi* was over twice as high for adults as for chicks (χ^2^_1, 146_ = 12.66, *P*<0.001; Table 1; Fig. 1). However, in this case, adults had a substantially lower infection intensity than chicks (*F*_1, 53_ = 20.51, *P*<0.0001; Table 1; Fig. 2).

**Table 1:** Comparisons of blood parasite *Haemoproteus nisi* and *Leucocytozoon toddi* infection prevalence and intensity in black sparrowhawks of different age and gender.

	Number of birds sampled	*Haemoproteus*		*Leucocytozoon*	
		Prevalence	Intensity	Prevalence	Intensity
**Adult**	44	0.72±0.07	4.31±1.19	0.59±0.07	1.42±0.64
Female	19	0.68±0.11	1.85±0.42	0.68±0.11	1.39±0.18
Male	25	0.76±0.09	6.00±1.90	0.52±0.10	1.46±0.18
Dark	32	0.76±0.08	2.84±0.74	0.58±0.09	1.39±0.16
Light	12	0.58±0.15	9.57±2.56	0.67±0.14	1.50±0.19
**Chick**	104	0.12±0.03	1.33±0.14	0.28±0.04	5.45±1.19
Female	39	0.15±0.06	1.17±0.17	0.36±0.08	4.29±1.71
Male	65	0.09±0.04	1.50±0.22	0.23±0.05	6.53±1.66

*P*revalence refers the proportion of birds infected with each parasite. Intensity values are the number of fields of view at 500× magnification where at least one infected cell was seen and relate only to infected individuals. Data are presented as means ±1 S.E.

### Sex differences in parasite infection

We found no difference in infection prevalence for *H. nisi* between sexes for either adults (χ^2^_1, 42_ = 0.31, *P* = 0.58; Table 1; Fig. 1) or chicks (*F*_1, 102_ = 0.49, *P* = 0.48; Table 1; Fig. 1). Likewise, no differences were found in infection prevalence for *L. toddi* between the sexes for adults (χ^2^_1, 42_ = 1.21, *P* = 0.27; Table 1; Fig. 1) or chicks (*F*_1, 102_ = 2.81; Table 1; *P* = 0.10; Fig. 1). However, among adults infected with *H. nisi*, males had a greater infection intensity than females (*F*_1, 30_ = 6.58, *P* = 0.006; Table 1; Fig. 2). *F*or *L. toddi* no differences in infection intensity was found between the sexes for either adults (*F*_1, 24_ = 0.10, *P* = 0.75; Table 1; Fig. 2) or chicks (*F*_1, 27_ = 0.84, *P* = 0.54; Table 1 Fig. 2).

### Relationship between adult morph and parasite infections

**Figure 3:**
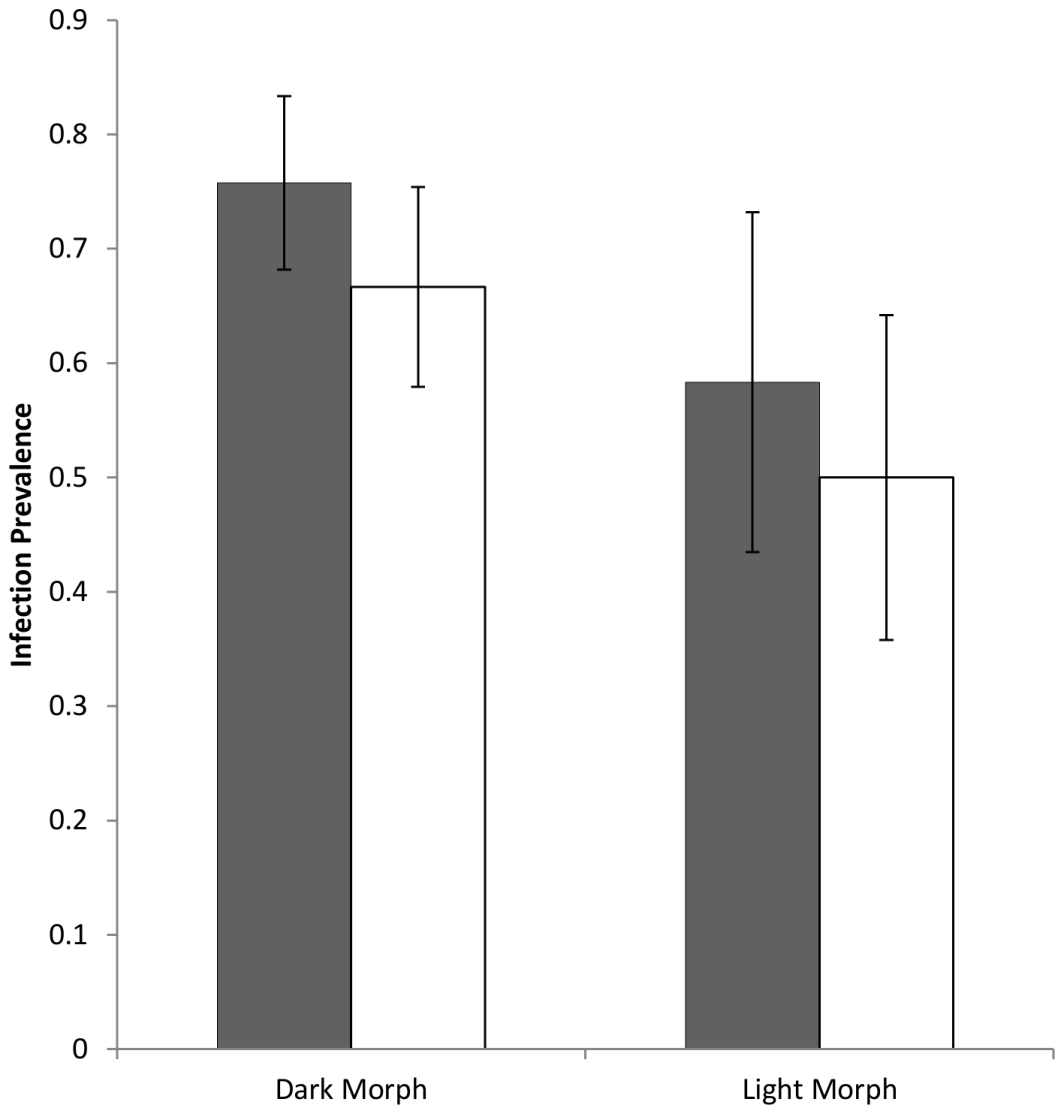
Differences in infection prevalence for the *Haemoproteus* (dark bars) and *Leucocytozoon* (light bars) blood parasites in the dark and light morphs of adult black sparrowhawks.

**Figure 4:**
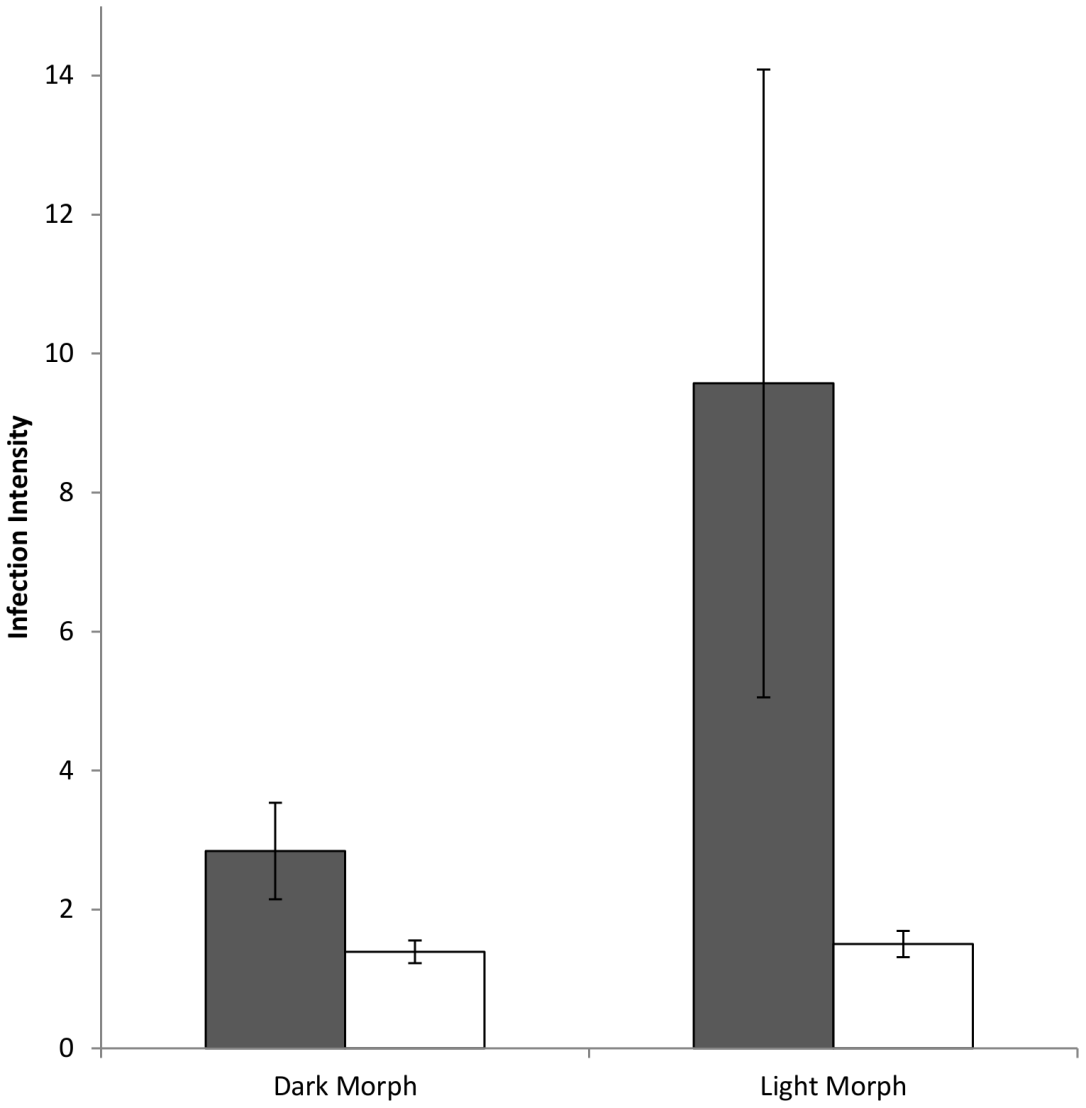
Differences in infection intensity for the *Haemoproteus* (dark bars) and *Leucocytozoon* (light bars) blood parasites in the dark and light morphs of adult black sparrowhawks.

*F*or adult birds, we found no difference in infection prevalence between the morphs. This was true for both *H. nisi* (χ^2^_1, 42_ = 1.64, *P* = 0.20; Table 1; Fig. 3) and for *L. toddi* (χ^2^_1, 42_ = 0.39, *P* = 0.53; Table 1; Fig. 3). However, for *H. nisi*, infection intensity was over three times higher for light birds than for dark birds, a difference which was highly significant (*F*_1, 30_ = 9.85, *P* = 0.001;Table 1; Fig. 4). This significant relationship remained even after controlling for the difference in infection intensity between the sexes (sex: *F*_1,29_ = 3.68, *P* = 0.055; morph: *F*_1,29_ = 6.60, *P* = 0.01). Infection intensity of *L. toddi* was much lower than for *H. nisi* (Fig. 4), and no difference was detected between the different morphs (*F*_1, 24_ = 0.07, *P* = 0.67; Table 1; Fig. 4).

### Relationship between parental morph and parasite infections in chicks

For chicks, we found no significant difference in the prevalence of either parasite depending on their maternal morph (*H. nisi*: *F*_1,57_ = 1.43, *P* = 0.23; *L. toddi*: *F*_1,45_ = 0.15, *P* = 0.69) or their paternal morph (*H. nisi*: *F*_1,100_ = 0.04, *P* = 0.23; *L. toddi*: *F*_1,59_ = 0.91, *P* = 0.34). Likewise, no difference for *L. toddi* infection intensity was found for chicks with different maternal morphs (GLMM estimates: dark: 5.00±2.77 light: 5.54±1.33, *F*_1, 27_ = 0.03, *P* = 0.86) or paternal morphs (GLMM estimates: dark: 4.6±1.07 light: 10.50±5.77, *F*_1, 23_ = 1.87, *P* = 0.18)

## Discussion

The results from this study provide support for our hypothesis that dark morph black sparrowhawks may have a selective immunological advantage against *Haemoproteus* parasites in our study area. We found support for our prediction for one of the parasites, with dark morphs adults having a lower infection intensity, although not prevalence, of *Haemoproteus nisi* than light morphs. Overall, infection intensity was far lower for *Leucocytozoon toddi* than *H. nisi*, and we found no difference in infection intensity or prevalence of *L. toddi* between the morphs. The difference between infection intensity and prevalence found in this study are strikingly similar to the other two studies that have compared haemoparasite prevalence and intensity between the adult morphs of birds. Both [17], on their study of feral pigeons, and [16], on their study of tawny owls, found that infection intensity, but not prevalence, differed between morphs, with darker pigeon morphs and greyer tawny owl morphs having lower infection intensity. Previous studies on *Haemoproteus* spp. have suggested that it is the intensity of parasite infection rather than its presence *per se* which is likely to have the greatest fitness consequence for an infected individual [40], [41]. Thus individuals may be able to cope with mild infection, whereas high levels of infection by *Haemoproteus spp* can cause severe morbidity in their avian hosts through direct tissue damage [40]. These results therefore emphasise the need to examine both measures of parasite intensity and prevalence when exploring this issue, which has not always the case (e.g. [19]).

If dark morphs are able to mount a better immune response to haemoparasites, it is not clear why infection intensity of *L. toddi* were not similarly reduced in dark morphs. Perhaps the lower levels of infection of *Leucocytozoon* compared with *Haemoproteus* (*L. toddi* 1.42±0.64 Vs. *H. nisi* 4.31±1.19) offers some explanation and may have meant that any potential for a stronger immune response by dark birds was never initiated. Interestingly, the only other study to examine differential parasitaemia of these two parasites between avian morphs also found differences in *Haemoproteus* infection intensity, but not *Leucocytozoon* infection intensity [16]. Similarly, [42] found no difference in *Leucocytozoon* infection between different morphs of tawny owls.

The lack of difference in infection prevalence for both parasites between adult morphs suggests that both morphs are equally exposed to these malarial parasites. However, the difference in intensity of the *H. nisi* infection suggests that dark morph birds are better able to mount an immune response to this parasite. Difference in intensity, rather than prevalence, suggests that the difference between morphs is unlikely to be due to morph-specific habitat selection but more likely from the improved ability of the dark morph to mount a stronger immune response. Since phagocytosing melanocytes confront pathogens, the greater melanin content of the dark morphs might aid their pathogen fighting capability [18]. Thus it is possible that differences in *Haemoproteus* infection pressure in different geographical regions is the selective agent behind the differences in morph ratios across regions, with a selective advantage for dark birds in our region, due to their ability to better resist heavy levels of parasitaemia of this parasite. The high prevalence of *H. nisi* (70%) in adult birds, and the rapid transmission rates as indicated by the many 3 week old chicks infected, suggests that this is a common parasite in black sparrowhawks in this region. Since *Haemoproteus* infections have known detrimental effects on reproductive success and survival in birds [30], [31], this parasite has the potential to be a strong selective force.

There are two, non-mutually exclusive, possible explanations as to why parasites in our study area may present a strong selective pressure driving traits associated with their resistance in this population relative to elsewhere in their range. Firstly, exposure to these parasites (parasite exposure hypothesis) may be greater due to vectors being more abundant in these wetter environments [31]. For example, vector abundance for *Haemoproteus* (Diptera: *Ceratopoginidae*) and *Leucocytozoon* (Diptera: *simuliidae*) are known to co-vary with rainfall [43]–[45]. Hence it is logical that wetter regions (such as the Cape Peninsula in the South African context) would encourage vector breeding, and that the transmission risk of these haematozoan parasites would be higher in such regions. Alternatively, breeding in these wetter regions may require more effort than breeding in drier regions and this increased effort may compromise the immune function (immune suppression hypothesis), leading to increased parasitic infections. Haemoparasite infections, including *Haemoproteus* infection intensity, have been shown in multiple experiments to rise with increased reproductive effort in birds [46]. A number of studies on other raptors have shown that levels of rainfall during the breeding season can strongly influence provisioning rates and subsequent breeding success [37], [47], [48]. Immune suppression caused by increased reproductive effort may be an important mechanism mediating the life-history cost of reproduction [46]. More stressful hunting conditions may lead to greater reproductive effort and a corresponding suppression of immune functions [49]. This may increase susceptibility to chronic infections and thus increase the requirement to invest in other forms of protection from parasites, such as the increased levels of melanin in darker morph birds [6].

Previous research on black sparrowhawks in the Cape Peninsula found a difference in the proportion of dark morph between the sexes, with a higher frequency of dark males (83%) than females (68%) and a tendency for dark morph males to have less white plumage compared to dark morph females [24]. If dark plumage helps individuals to resist chronic parasite infection, then this result might indicate that males are under even greater pressure during the reproductive period from parasites and consequently greater selective pressure for dark plumage. Numerous studies have shown a link between testosterone and immune suppression [50]–[52] with males often exhibiting inferior immuno-competence to females in a range of vertebrates [53], [54] including several bird species [55] [57]. In many cases this is reflected in higher parasite loads in males particularly when parasites are most prevalent [56]. In support of this, we found infection intensity for *H. nisi* was higher in males than females. This has important implications in our study species, and particularly for males, as an increased immuno-competency, believed to be possessed by darker morph birds, may be necessary for individuals to cope with their parasite loads. Selection in favour of dark morphs may therefore be strongest for male black sparrowhawks, particularly during the breeding periods, where they invest more effort into hunting and providing for their offspring and females, and when parasites are likely to be most prevalent (i.e. in the wet breeding season).

Our study is the first to examine differences in haemoparasitic infection intensity levels between different adult morphs in any raptor species. Other studies exploring the issue have examined the difference in parasite infection intensity [18], or immune response [6] of chicks in relation to their morph or their parental morphs. We found no difference in prevalence or infection intensity, for either parasite, between chicks with different parental morphs. There are two possible explanations for that finding; either chicks were sampled too young for any difference between the morphs to have become established. Alternatively, because the expression of the different morphs (unlike most other raptors) occurs in this species only once it is in adult plumage the higher melanin levels which may assist in the immune system might not be available to the chicks. This strategy appears to conform most closely with the immune suppression hypothesis, there are presumably costs associated with melanin rich plumage and delaying the development of melanin rich plumage until adulthood could be an adaptive strategy. Then its production is limited only to the reproductive life stage when infection intensities may be highest and the immunological benefits of being dark outweigh the costs associated with melanin rich plumage. Costs for dark birds are unlikely to be based purely on production costs, since many studies have shown that for vertebrates melanin synthesis is not a costly exercise [58]. These costs may instead come in the form of some comprised abilities (e.g. reduced foraging success within certain habitat type or on certain prey species) [59] or may be linked to other behavioural traits (e.g. aggression) [60]–[62] or to other physiological costs (e.g. Antioxidant machinery) [63].

To further explore whether the higher frequency of dark morphs in our region was driven by parasite exposure or immune suppression, we would need to compare parasite infection prevalence and intensity between morphs in our wet winter study area with other populations in the rest of the historical range (where the light morph predominates) which experience minimal rain during the winter breeding season. If the exposure hypothesis is correct, we would predict a lower *H. nisi* prevalence in these other areas due to a lower abundance of vectors. In contrast, if the immune suppression hypothesis is correct, we would expect similar *H. nisi* prevalence, but a higher infection intensity in birds of the southwest since birds breeding in more stressful wet conditions would have suppressed immune systems. For both hypotheses, we would predict that in these dry winter areas there would be little or no difference in the parasite prevalence or intensity between dark and light morphs. Additionally, we also need to establish the fitness consequence of high *Haemoproteus* burdens for black sparrowhawks in our region.

To conclude, this study supports the hypothesis that genes which code for darker plumage of polymorphic bird species may also play a role in the immune function, in particular with resistance to higher infection intensity by *Haemoproteus* parasites. Although relatively few studies have explored this issue, there does appear to be some consistencies in their findings. That these studies come from different bird species from different families suggests that there may be some generality to this finding for other polymorphic species. The lack of large scale studies and meta-analyses exploring the general patterns of *Haemoproteus* infection between bird species [64] hinders our understanding of the role that *Haemoproteus* infections may have in driving the evolution of polymorphism in birds. Such an analysis might therefore be particularly revealing to explore whether polymorphic species or families with high rates of polymorphism may have higher prevalence and specifically higher intensity levels of *Haemoproteus* infections.
